# Current update on herbal sources of antithrombotic activity—a comprehensive review

**DOI:** 10.1186/s43162-021-00090-9

**Published:** 2022-03-07

**Authors:** Bhavani Subramani, P. Sathiyarajeswaran

**Affiliations:** Siddha Central Research Institute (SCRI), Central Council for Research in Siddha (CCRS), Arumbakkam, Chennai, Tamilnadu 600106 India

**Keywords:** Antiplatelet, Antithrombotic, Anticoagulant, Herbal medicine, Phytochemicals, Secondary metabolites, Alkaloids, Saponins, Flavonoids, Coumarins

## Abstract

**Background:**

Herbs are commonly used to treat cardiovascular diseases in various traditional medicine. On the other hand, herb-drug interactions are most commonly encountered with conventional antiplatelet and anticoagulant drug prescriptions. This review presents a compilation of plants investigated for antiplatelet and anticoagulation recently and enumerates their possible lead compounds responsible for its action for paving further drug discovery and knowledge update.

**Main body of the abstract:**

Information about the herbs was withdrawn from the PubMed database of the previous 5 years. We also hand-searched the bibliography of relevant articles for the acquisition of additional information. About 72 herbal sources were identified with the effect of antiplatelet activity, antithrombotic activity, and anticoagulant activity. Bioactive compounds and various secondary metabolites responsible for it, such as alkaloids, saponins, flavonoids, coumarins, polyphenols, furan derivatives, iridoid glycosides, sesquiterpenes, aporphine compounds, were reported.

**Conclusion:**

Newer pharmacological moieties are needed to prevent or reduce the adverse effects of current anti-thrombotic agents and to improve the safety of patients and cost-effectiveness.

## Background

Cardiovascular disease (CVD) due to thrombosis comprises coronary artery disease (CAD), stroke, hypertension, peripheral arterial disease (PAD), venous-thrombo-embolic disease (VTE) [1]. As per the National Health and Nutrition Examination Survey (NHANES) 2013–2016, the prevalence of Coronary heart disease (CHD) in the USA was estimated as 18.2 million in > 20 years of age with more risk among males than females, whereas the prevalence of ischaemic stroke was 67.6 million and that of hemorrhagic stroke was 15.3 million [2]. CVD and stroke accounted for 14% of the total expenditure in 2014–2015, more than any diagnostic group results in immense health and economic burden in the USA globally. The AHA’s 2020 Impact Goals are to improve the cardiovascular health of all Americans by 20% while reducing deaths attributable to CVD and stroke by 20% [1].

Currently, witnessing an unprecedented pandemic, the coronavirus disease 2019 (COVID-19), caused by severe acute respiratory syndrome coronavirus 2 (SARS Co-V-2), associated with a significant risk of thromboembolic complications due to hypercoagulability state of blood which is called as Covid-19 associated coagulopathy (CAC) [3]. Though prophylaxis anti-coagulants were administered, the incidence of VTE complications was reported in two-thirds of ICU cases [4] and developed life-threatening thrombotic complications followed by Acute respiratory distress syndrome (ARDS) [5]. Venous thromboembolism (VTE), a major cardiovascular complication, was observed in about more than 20% of critically ill COVID-19 cases, particularly among critically ill viral pneumonia patients [4]. Histologically, significant thrombosis in small blood vessels and micro-vasculature of pulmonary and extra-pulmonary organs have been confirmed [6], widespread prevalence of deep vein thrombosis and pulmonary embolism, as well as microthrombi in the small pulmonary vessels in autopsy findings [7]. Several hypotheses on the mechanism of thrombosis in Covid-19 have been proposed and remain unclear.

### Antiplatelets and anti-coagulants

Thrombosis can be classified as arterial thrombosis and venous thrombosis although overlaps may be present. In general, pharmacologically two classes of drugs are used to prevent blood clots such as antiplatelets and anticoagulants [8]. Antiplatelets act by inhibition of platelet adhesion and activation and aggregation of thrombosis [9]. Thrombosis refers to the formation of platelet or fibrin aggregation in the lumen of the blood vessels or heart [10]. Anticoagulants prevent blood clot formation by interfering with proteins responsible for blood clotting or clotting factors [8]. Hypercoagulability is the state of increased tendency to the formation of thrombosis also triggering intracellular signalling for inflammation [10]. The use of antithrombotic medications remains the mainstay of treatment in cardiovascular and cerebrovascular disorders. Aspirin and clopidogrel were the commonly administered antiplatelet drugs to reduce recurrent ischaemic events in CAD and ischaemic stroke. Oral anticoagulants are prescribed for primary prevention and secondary prevention of venous thromboembolic disease [11] and as the best option in the prevention of stroke due to cardio-embolism in atrial fibrillation [12].

### Adverse drug reaction due to conventional antithrombotic drug regimen

Aspirin is prone to cause gastrointestinal side effects, hypersensitivity, hypo-responsiveness in some, and bleeding episodes [13]. Low-dose aspirin is commonly used as primary and secondary prevention of cardiovascular disease, which is associated with the risk of upper and lower gastro-intestinal tract lesions, particularly in the upper gastro-intestinal tract which may cause asymptomatic lesions to peptic ulcer bleeding and/or even death Li et al. [14].

Until recently, the vitamin K antagonists were the only oral anticoagulant agents available and warfarin remains the most commonly prescribed oral anticoagulation worldwide [15]. Warfarin has significant variability in dose-response across individuals and a narrow therapeutic window and intensive therapeutic monitoring are essential. When combined with low-dose aspirin, NSAIDs, or clopidogrel, warfarin acts cumulatively and the risk of bleeding is significantly increased [16] The risk of major bleeding associated with oral anti-coagulants ranges from 3.26 to 7.2% annually [11]. Both oral anticoagulation and antiplatelet therapies are essential in 20–30% of patients with co-existing atrial fibrillation (AF) and CAD, together posing a major risk of thrombotic complications [17]. Currently, in the management of patients with IHD and AF, include triple therapy TT (an anticoagulant plus 2antiplatelet drugs) and two types of dual therapy, DAPT (2 antiplatelet drugs) or DT (an anticoagulant plus a single antiplatelet drug) [18].

### Herbal resources and secondary metabolites

Herbs play an indispensable role in natural product discovery to meet the growing healthcare needs. Researchers screen herbal sources through reverse pharmacology and observational therapeutics to find novel compounds and harness the potential for future drug discovery. According to WHO (World Health Organization), about 80% of the World’s population depends on medicinal plants or herbs to fulfill their medicinal needs. Herbal medicines are a maximum part of complementary and alternative medicine and preferred treatment of people for various reasons such as ethnicity of use, family traditions, and past good experiences [19]. In this review, we have covered 72 herbs, their extracts, their secondary metabolites, and their pharmacological activities studied in both in vivo, ex vivo, and in vitro investigations. Acknowledging the growing significance of traditional medicine and usage, the WHO global report on traditional and complementary medicine 2019 states about the steps taken to promote the safety, quality, and effectiveness of traditional medicine by developing the WHO Traditional Medicine Strategy 2014–2023, in line with WHO Traditional Medicine Strategy (2002–2005). Healthcare professionals need to be aware of and monitor possible risks of concomitant medications of herbs with conventional medicine prescriptions if any [20].

## Methods

We conducted a PubMed search for the in-vitro and in vivo studies published between 2016 and 2020 till December using multiple combinations of keywords, including the following: “anti-thrombotic activity”, “antiplatelet activity”, “anti-coagulant”, “antiplatelet aggregation”, “anti-hyper-viscosemia”, “anti-aggregant”, “platelet agglutination inhibitor”, “platelet aggregation inhibitor”, “platelet targeted pharmacologic agents”, “antiplatelet adhesion”, “medicinal plants”, and “herbal sources”. We found 296 publications that were reviewed by two authors. The retrieved articles were examined to eliminate potential duplicates or overlapping data. We also hand-searched the references of relevant articles for the acquisition of additional information. We included only those studies published in peer-reviewed journals in the English language only. Finally, 26 manuscripts were considered for this review. The botanical names of all the plants enumerated below (Table 1) were verified referring to www.theplantlist.org.
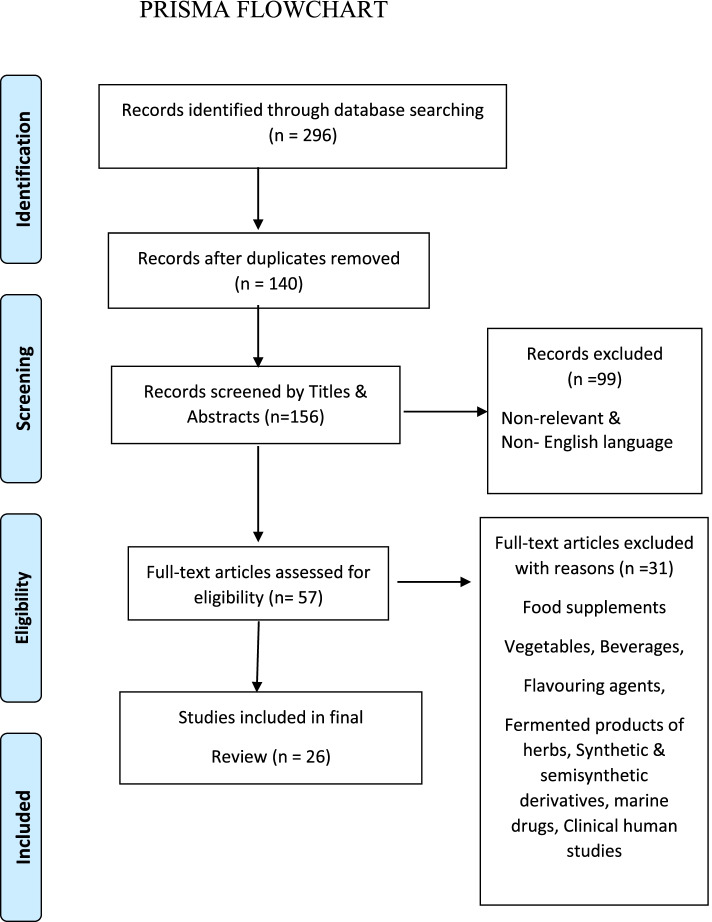

**Table 1:** List of herbal sources of antithrombotic and its phytoconstituents

Family	Botanical name	Parts used	Effect/activity	Phytochemicals	References
Apiaceae	*Angelica keiskei (Miq.) Koidz.*	Stem	Antithrombotic-anti-coagulant	Xanthoangelol B	[21]
Apiaceae	*Angelica sinensis (Oliv.) Diels*	Aerial parts	Anti-coagulant, antiplatelet	Z-Ligustilide	[22]
Malvaceae	*Abelmoschus manihot (L.) Medik*	Plant	Antiplatelet	Total flavone	[23]
Acanthaceae	*Andrographis paniculata (Burm.f.)Nees*	Plant	Antiplatelet	Diterpenoids	[24]
Liliaceae	*Anemarrhena asphodeloides Bunge*	Rhizomes	Antiplatelet; antithrombotic	Timosaponin A-III, timosaponin B-II, anemarsaponin B, steroidal glycosides	[25]
Apiaceae	*Apium graveolens Linn*	Seeds	Antithrombotic, antiplatelet	3-N-Butylphthalide (NBP)l-3-n-butylphthalide (NBP)	[26]
Amaranthaceae	*Achyranthes bidentatata Blume*	Plant	Anticoagulant	Polysaccharides	[27]
Liliaceae	*Allium sativum L.*	Cloves	Antiplatelet	Allicin, adenosine,paraffinic polysulfides	[28]
Sapindaceae	*Aesculus hippocastanum L.*	Bark	Anticoagulant	Aescin (coumarin)	[29]
Berberidaceae	*Berberis vulgaris L.*	Plant	Antiplatelet	Berberine	[30]
Myrtaceae	*Campomanesia xanthocarpa (Mart.) O.Berg*	Leaf	Antithrombotic,antiplatelet	Flavonoids	[31]
Cyperaceae	*Cyperus rotundus L.*	Tuber	Antiplatelet	(+)-nootkatone(sesquiterpenoid)	[32]
Cornaceae	*Cornus mas L*	Dried fruits	Anticoagulant	Anthocyanins, polyphenols	[33]
Lauraceae	*Cassytha filiformis L.*	Fresh herb	Antiplatelet	Aporphinoid alkaloids	[34]
Zingiberaceae	*Curcuma aromatica Salisb.*	Rhizome	Antiplatelet	Curcumin	[35]
Asteraceae	*Chrysanthemum indicum L.*	Flowers	Antiplatelet	Chlorogenic acid	[36]
Lauraceae	*Cinnamomum cassia Nees.*	Bark and twigs	Antiplatelet	Eugenol, amygdalactone, cinnamic alcohol, 2-hydroxycinnamaldehyde, 2-methoxycinnamaldehyde, coniferaldehyde	[37]
Rutaceae	*Citrus hassaku Yu.Tanaka*	Fruits	Antiplatelet	Prunin	[38]
Ranunculaceae	*Coptis chinensis Franch.*	Rhizome	Antiplatelet	Berberine	[39]
Compositae	*Carthamus tinctorius L.*	Plant	Antithrombotic	Hydroxysafflor yellow A	[40]
Leguminosae	*Caesalpinia sappan L.*	Heartwood	Antiplatelet	Brazilin	[41]
Zingiberaceae	*Curcuma longa L.*	Rhizome	Antiplatelet, anticoagulant, antithrombotic	Ar-turmerone, curcumin	[42, 43]
Moraceae	*Cudrania tricuspidata Bureau*	Roots	Antiplatelet	Cudratricusxanthone A (CTXA)	[44]
Lamiaceae	*Callicarpa nudiflora Hook. & Arn.*	Leaves	Antiplatelet	Triterpenoids	[45]
Apiaceae	*Centella asiatica L. (Urb).*	Herb	Antiplatelet	Caffeoyl quinic acid compounds	[46]
Fabaceae (Leguminosae	*Dalbergia odorifera T. Chen*	Heartwood	Antiplatelet	Sesquiterpenes	[47]
Dioscoreaceae	*Dioscorea zingiberensis C.H. Wright*	Rhizome	Antithrombotic, anticoagulant, antiplatelet	Dioscin-steroidal saponins	[48, 49]
Ebenaceae	*Diospyros kaki Thunb.*	Leaves, fruits	Anticoagulant, antithrombotic	Diosmin (diosmetin 7-O-rutinoside), a disaccharide derivative	[50]
Euphorbiaceae	*Euphorbia neriifolia L.*	Roots, leaves	Antithrombotic	Flavonoids, polyphenols	[51]
Rutaceae	*Evodia rutaecarpa A.Juss.*	Dried unripened fruit	Antiplatelet	Rutaecarpine	[52]
Asteraceae	*Erigeron canadensis L.*	Whole plant	Anticoagulant, antiplatelet	Polyphenolic polysaccharide	[53]
Ginkgoaceae	*Ginkgo biloba L.*	Leaf	Antiplatelet activity	Ginkgolides A, B, and C	[54]
Leguminosae	*Glycyrrhiza uralensis*	Rhizome	Antithrombotic	Isotrifoliol	[55]
Himantandraceae	*Galbulimima baccata F.M.Bailey*	Bark	Antithrombotic	Galbulimima alkaloids-himbacine	[56]
Saururaceae	*Houttuynia cordata*	Plant	Antiplatelet	Alkaloids	[57]
Hernandiaceae	*Hernandia nymphaefolia J.Presl.*	Trunk bark	Antiplatelet	Aporphine compounds	[58]
Hernandiaceae	*Illigera luzonensis Merr*	Roots	Antiplatelet	Aporphine alkaloids	[59]
Aquifoliaceae	*Ilex paraguariensis A.St.*	Fruits	Antithrombotic, antiplatelet	Chikusetsusaponin IVa	[60]
Lamiaceae	*Leonurus sibiricus *	aerial parts	antiplatelet	Leonurine	[61]
Caprifoliacea	*Lonicera japonica Thunb.*	plant	antiplatelet	Protocatechuic acid	[62]
Lamiaceaeae	*Lycopus lucidus Turcz.*	plant	antiplatelet	-	[63]
Asparagaceae	*Liriope muscari L.H.Bailey.*	plant	anti‐thrombotic	D39, a natural saponin	[64]
Lauraceae	*Lindera obtusiloba Blume*	Leaf	antiplatelet, antithrombotic	quercitrin and afzelin	[65]
Rutaceae	*Melicope semecarpifolia Merr.*	root bark	antiplatelet	quinoline alkaloids,	[66]
Magnoliaceae	*Magnolia officinalis*	Bark	antiplatelet	Magnolol,honokiol	[67]
Nelumbonaceae	*Nelumbo nucifera Gaertn.*	fruits ;whole plant	anti-coagulant; antithrombotic	neferine, alkaloid; flavonoids in hydroalcoholic extract respectively	[68]
Lamiaceae	*Origanum majorana L.*	plant	antiplatelet	hydroquinone-D-glucopyranoside (Coumarin )	[69]
Oleaceae	*Osmanthus fragrans Lour.*	seeds	antiplatelet	secoiridoid glucoside	[70]
Araliaceae	*Panax ginseng Meyer*	root	antiplatelet	Ginsenoside Rg1, Ginsenoside Rg3, Ginsenoside Rp4.Ginsenoside Ro (an oleanane-type saponin	
Piperaceae	*Piper longum L.*	Dried fruits	antiplatelet	piperlongumine,a pyridone alkaloid	[71]
Paeoniaceae	*Paeonia suffruticosa*	dried root bark	antiplatelet	-	[72]
Paeoniaceae	*Paeonia lactiflora Pall.*	plant	antiplatelet and anti-coagulant	Paeoniflorin, Benzoylpaeoniflorin, Benzoyloxypaeoniflorin, Methyl gallate, Catechin, Paeoniflorigenone, Galloylpaeoniflorin, Daucosterol	[72]
Araliaceae	*Panax bipinnatifidus Seem.*	Roots	antithrombotic,antiplatelet	saponins	[73]
Annonaceae	*Rollinia mucosa Jacq.*	stems	antiplatelet	N-methoxycarbonyl aporphine alkaloids,romucosine A (1), romucosine B (2), romucosine C (3), andromucosine D (4	[74]
Apocynaceae	*Rauwolfia serpentina Benth.*	roots	antiplatelet	Ajmaline	[75]
Rutacaeae	*Ruta graveolens L.*	root and aerial parts	antiplatelet	The quinoline alkaloid graveolinine	[76]
Anacardiaceae	*Rhus verniciflua (Syn.Toxicodendron vernicifluum)*	herb	antiplatelet	Isomaltol, Pentagalloyl glucose	[77]
Polygonaceae	*Rheum palmatum L.*	aerial parts	antiplatelet	Two stilbenes- trans-resveratrol-3-O-β-d-glucopyranosid (I) and rhaponticin (II)	[78]
Scrophulariaceae	*Rehmannia glutinosa (Gaertn.)*	dried roots	antiplatelet	furan derivatives	[79]
Rosaceae	*Spiraea japonica L.*	roots	antiplatelet	atisine-type diterpenoid alkaloids	[80]
Lamiaceae	*Scutellaria baicalensis Georgi.*	root	anti-platelet, anticoagulant	Baicalin	[81]
Leguminosae	*Spatholobus suberectus Dunn.*	stem	antiplatelet	daidzein and genistein	[82]
Fabaceae	*Sophora japonica L.*	plant	antiplatelet	flavonoids	[83]
Selaginellaceae	*Selaginella tamariscina (P. Beauv.) Spring*	herb	anti-coagulant	dihydrocaffeic acid & amentoflavone	[84]
Typhaceae	*Sparganium stoloniferum Buch.*	plant	antiplatelet, antithrombotic	flavonoids	[9]
Labiateae	*Salvia miltiorrhiza*	Root	antiplatelet	15,16-dihydrotanshinone I, Tanshinone I, Tanshinone IIA, Cryptotanshinone, Danshensu, Salvianolic acid B	[85]
Sapindaceae	*Sapindus mukorossi Gaertn.*	Galls	antiplatelet	Sapinmusaponins F-J; Sapinmusaponins Q and R (1–50 µM) respectively	[86]
Asteraceae	*Silybum marianum (L.) Gaertn.*	Seeds,fruits	antiplatelet activity	Silymarin( flavonolignans)	[87]
Rosaceae	*Spiraea japonica L.*	roots	antiplatelet	spiramine C1	[80]
Violaceae	*Viola yedoensis Makino*	whole plants	anticoagulant	dicoumarins: dimeresculetin, euphorbetin, esculetin	[88]
Melanthiaceae	*Veratrum dahuricum (Turcz.) O.Loes.*	rhizomes	antiplatelet	Veratroylgermine-steroidal alkaloid	[89]
Zingiberaceae	*Zingiber officinale Roscoe*	rhizome	antiplatelet	Gingerol, paradol	[90]

### Mechanism of antiplatelet and anticoagulant activity of herbs

Plant-derived compounds such as alkaloids, anthraquinones, coumarins, flavonoids, xanthones, Lignans, saponins, stilbenes, etc. were found to affect platelet aggregation activity Werner Cordier et al. [91]. Inhibition of platelet adhesion or chemical mediators for activation of platelet function is the common potential of herbs for its antiplatelet activity. Various mechanisms had been postulated such as inhibition of ADP-induced platelet aggregation, inhibition of the arachidonic acid pathway, thereby inhibiting biosynthesis of thromboxane A2; plants containing lignans, xanthones, sesquiterpenes, flavonoids affect coagulation by inhibiting platelet-activating factor (PAF), or PAF receptor antagonists, inhibiting the factor X on the coagulation cascade. Plants containing the coumarin class of compounds antagonise vitamin K and prevent coagulation. Few naturally occurring compounds contain fibrinolytics which may activate plasminogen and affect coagulation. Phytochemicals that inhibit the CYP3A4, CYP2C9, and CYP1A2 metabolism were potent to affect coagulation Leite et al. [92]. Herbs identified in this review were listed with possible mechanisms of action responsible for their pharmacological activity in Table 2.

**Table 2:** List of herbal sources with mechanisms of its pharmacological action

Botanical name	Mechanism of action
*Angelica keiskei (Miq.) Koidz.*	Inhibit platelet aggregation
*Angelica sinensis (Oliv.) Diels*	Inhibit platelet aggregation
*Abelmoschus manihot (L.) Medik*	Inhibit platelet aggregation
*Andrographis paniculata (Burm.f.) Nees*	Inhibit platelet aggregation
*Anemarrhena asphodeloides Bunge*	Inhibit ADP-induced platelet aggregation
*Apium graveolens Linn*	Inhibit platelet aggregation
*Achyranthes bidentatata Blume*	Prolonged coagulation time
*Allium sativum L.*	Inhibit platelet aggregation
*Aesculus hippocastanum L.*	Preventing oxidative damage of fibrinogen & moderate antiplatelet aggregation activity
*Berberis vulgaris L.*	Inhibit platelet aggregation
*Campomanesia xanthocarpa (Mart.) O. Berg*	Inhibit platelet aggregation, fibrinolytic activity
*Cyperus rotundus L.*	Inhibit collagen-, thrombin-, and AA-induced platelet aggregation
*Cornus mas L*	Inhibit platelet aggregation
*Cassytha filiformis L.*	Inhibit platelet aggregation
*Curcuma aromatica Salisb.*	Inhibit AA-, collagen-, & ADP-induced platelet aggregation
*Chrysanthemum indicum L.*	Inhibit platelet aggregation
*Cinnamomum cassia Nees.*	Inhibit platelet aggregation
*Citrus hassaku Yu. Tanaka*	Inhibit platelet aggregation
*Coptis chinensis Franch.*	Inhibited thromboxane synthesis
*Carthamus tinctorius L.*	Inhibited thromboxane synthesis
*Caesalpinia sappan L.*	Inhibited collagen-induced platelet aggregation
*Curcuma longa L.*	Inhibit platelet aggregation
*Cudrania tricuspidata Bureau*	Inhibit platelet aggregation, inhibited thrombin production
*Callicarpa nudiflora Hook. & Arn.*	Antiplatelet aggregation
*Centella asiatica L. (Urb).*	Inhibition of platelet activation and coagulation
*Dalbergia odorifera T. Chen*	Inhibit platelet aggregation
*Dioscorea zingiberensis C.H. Wright*	Antithrombotic
*Diospyros kaki Thunb.*	Inhibited thrombin-catalysed fibrin formation
*Euphorbia neriifolia L.*	Prolonged bleeding time & clotting time
*Evodia rutaecarpa A. Juss.*	Prolonged bleeding time, antiplatelet aggregation
*Erigeron canadensis L.*	Inhibited thrombin
*Ginkgo biloba L.*	Inhibit platelet aggregation
*Glycyrrhiza uralensis*	Antithrombotic
*Galbulimima baccata F.M. Bailey*	Inhibit platelet aggregation
*Houttuynia cordata*	Antiplatelet aggregation
*Hernandia nymphaefolia J. Presl.*	Antiplatelet aggregation
*Illigera luzonensis Merr*	Antiplatelet aggregation
*Ilex paraguariensis A.St.*	Inhibits fibrinogen & platelet aggregation
*Leonurus sibiricus*	Antiplatelet aggregation
*Lonicera japonica Thunb.*	Antiplatelet aggregation
*Lycopus lucidus Turcz.*	Inhibit aggregation of red blood cells
*Liriope muscari L.H. Bailey.*	Inhibit thrombosis
*Lindera obtusiloba Blume*	Inhibit platelet aggregation & collagen-induced thromboxane production
*Melicope semecarpifolia Merr.*	Antiplatelet aggregation
*Magnolia officinalis*	Antiplatelet aggregation
*Nelumbo nucifera Gaertn.*	Inhibitory effect on platelet activation, adhesion & aggregation, and thromboxane A2 formation
*Origanum majorana L.*	Inhibition of platelet adhesion & aggregation
*Osmanthus fragrans Lour.*	Inhibit platelet aggregation
*Panax ginseng Meyer*	Antiplatelet aggregation
*Piper longum L.*	Inhibit AA-, collagen-, & PAF-induced platelet aggregation
*Paeonia suffruticosa*	Inhibit platelet aggregation & blood coagulation
*Paeonia lactiflora Pall.*	Inhibit platelet aggregation & blood coagulation
*Panax bipinnatifidus Seem.*	Inhibit platelet aggregation & prolonged aPTT
*Rollinia mucosa Jacq.*	Inhibit platelet aggregation
*Rauwolfia serpentina Benth.*	Inhibition of platelet-activating factor
*Ruta graveolens L.*	Antiplatelet aggregation
*Rhus verniciflua (Syn.Toxicodendron vernicifluum)*	Antiplatelet aggregation
*Rheum palmatum L.*	Antiplatelet aggregation
*Rehmannia glutinosa (Gaertn.)*	Antiplatelet aggregation
*Spiraea japonica L.*	Antiplatelet aggregation
*Scutellaria baicalensis Georgi.*	Inhibited fibrin polymerization and platelet function, prolonged aPTT, PT, and production of thrombin
*Spatholobus suberectus Dunn.*	Inhibition of fibrinogen binding
*Sophora japonica L.*	Antiplatelet aggregation
*Selaginella tamariscina (P. Beauv.) Spring*	Antiplatelet aggregation & increased fibrinogen content
*Sparganium stoloniferum Buch.*	Antiplatelet aggregation
*Salvia miltiorrhiza*	Inhibit platelet aggregation
*Sapindus mukorossi Gaertn.*	Antiplatelet aggregation
*Silybum marianum (L.) Gaertn.*	Antiplatelet aggregation
	Antiplatelet aggregation
*Viola yedoensis Makino*	Prolonged aPTT, PT
*Veratrum dahuricum (Turcz.) O. Loes.*	Inhibit AA-induced platelet aggregation
*Zingiber officinale Roscoe*	Antiplatelet aggregation

*ADP* adenosine di-phosphate, *AA* arachidonic acid, *PAF* platelet-activating factor, *aPTT* activated partial thromboplastin time, *PT* prothrombin time

### Herb-drug interaction types and mechanism

Among older adults, concomitant herbal medicine use along with prescription drugs had been reported as 5.3 to 88.3% in a systematic review as potential cause of herbal-drug interaction Agbabiaka et al. [93]. Herb-drug interactions (HDI) may be either due to pharmacokinetic or pharmacodynamic interactions which affects the safety and efficacy of the treatment. Pharmacokinetic interactions affect the absorption, distribution, metabolism, and excretion of drugs which in turn results in a change in drug concentration in body fluids Lee et al. [94]. Various mechanism has been postulated for the altered drug concentration such as induction or inhibition of hepatic and intestinal drug-metabolizing enzymes such as cytochrome P450, UDP-glucorynyl transferase, and carrier proteins such as P-glycoprotein was suggested Kahrman et al. [95]. While pharmacodynamic interactions are related to the pharmacological activity of the interacting agents which may be synergistic or additive resulting in toxicities or antagonistic causing treatment failure Izzo [96].

### Herbal drug interaction with aspirin, clopidogrel, and warfarin

Few frequently reported herbs, with its commonly used therapeutic indications (Table 3), and drug interactions with conventional anti-thrombotic medicines were enumerated with increased risk of bleeding as per current evidence (Tables 4, 5, and 6) and types of herb-drug interaction of few herbs are summarised (Table 7).

**Table 3:** Common therapeutic indication of herbs

Herbs	Main uses of herb	Reference
*Angelica sinensis (Oliv.) Diels*	Promoting circulation	Lu et al. [97]
*Andrographis paniculata (Burm.f.) Nees*	Myocardial ischaemia, fever, respiratory infections	Zhang et al. [6]
*Apium graveolens Linn*	Hepatic and spleen disorders, brain disorders, sleep disturbances	Al-Asmari et al. [98]
*Allium sativum L.*	Hypercholesterolaemia	Izzo et al. [96]
*Aesculus hippocastanum L.*	Anti-inflammatory, venotonic	Sparg et al. [29]
*Carthamus tinctorius L.*	Chest pain, traumatic injuries	Lim et al. [99]
*Curcuma longa L.*	Chest pain, amenorrhoea	Lim et al. [99]
*Centella asiatica L. (Urb).*	Improving memory	Satake et al. [46]
*Ginkgo biloba L.*	CVD, angina, cerebral vasospasm, hypertension	Lim et al. [99]
*Panax ginseng Meyer*	Enhancing immunity, cognitive impairment	Kim et al. [100]; Lim et al. [99]
*Salvia miltiorrhiza*	Cardiovascular and cerebrovascular symptoms	Kim et al. [100]
*Silybum marianum (L.) Gaertn.*	Liver and gallbladder disorders	Gurley et al. [101]
*Zingiber officinale Roscoe*	Anti-bacterial, anti-ulcer	Mohd Nor et al. [102]

**Table 4:** List of herb-aspirin interaction causing increased risk of bleeding

Botanical name	Herb-aspirin interaction (references)
*Angelica sinensis (Oliv.) Diels*	Xiao et al. [103]
*Carthamus tinctorius L.*	Lim et al. [99]
*Curcuma longa L.*	Hu and Wang [104]
*Ginkgo biloba L.*	Hu and Wang [104]
*Panax ginseng Meyer*	Hu and Wang [104]
*Salvia miltiorrhiza*	Hu and Wang [104]; Xiao et al. [103]

**Table 5:** List of herb-clopidogrel interaction causing increased risk of bleeding

Botanical name	Herb-clopidogrel interaction (references)
*Angelica sinensis (Oliv.) Diels*	Xiao et al. [103]
*Carthamus tinctorius L.*	Lim et al. [99]
*Curcuma longa L.*	Lim et al. [99]
*Ginkgo biloba L.*	Lim et al. [99]
*Panax ginseng Meyer*	Lim et al. [99]
*Salvia miltiorrhiza*	Lim et al. [99]; Xiao et al. [103]

**Table 6:** List of herb-warfarin interaction causing increased risk of bleeding

Botanical name	Herb-warfarin interaction (references)
*Angelica sinensis (Oliv.) Diels*	Leite et al. [92]; Ge et al. [105]; Akram and Rashid [106]; Leite et al. [107]
*Andrographis paniculata (Burm.f.) Nees*	Leite et al. [107]
*Apium graveolens Linn*	Akram and Rashid [106]
*Allium sativum L.*	Leite et al. [92]; Leite et al. [107]
*Aesculus hippocastanum L.*	Leite et al. [107]
*Carthamus tinctorius L.*	Leite et al. [107]
*Curcuma longa L.*	Leite et al. [92]; Ge et al. [105]; Akram and Rashid [106]; Shaikh et al. [108]; Leite et al. [107]
*Centella asiatica L. (Urb).*	Leite et al. [107]
*Ginkgo biloba L.*	Leite et al. [92]; Ge et al. [105]; Akram and Rashid [106]; Shaikh et al. [108]; Leite et al. [107]
*Panax ginseng Meyer*	Akram and Rashid [106]; Shaikh et al. [108]
*Salvia miltiorrhiza*	Akram and Rashid [106]; Shaikh et al. [108]
*Silybum marianum (L.) Gaertn.*	Leite et al. [107]
*Zingiber officinale Roscoe*	Leite et al. [92]; Ge et al. [105]; Leite et al. [107]

**Table 7:** Types of herb-drug interaction in herbs

Herb	Warfarin	Aspirin	Clopidogrel
*Angelica sinensis (Oliv.) Diels*	(A) COX-inhibitor [Hu et al. 2005]. Inhibits CYP1A2 & CYP3A4 Leite et al. [92]	(A) Inhibition of rCyp2c11 & carboxylesterase activities Xiao et al. [103]	(A) Inhibition of rCyp2c11 & carboxylesterase activities Xiao et al. [103]
*Allium sativum L.*	(A) Intereferes with metabolizing enzymes Ge et al. [105]; (B) additive effect [Hu et al. 2005]; (B) PAF inhibitor Ge et al. [105]; (A) inhibits CYP3A4 Leite et al. [92]	–	–
*Aesculus hippocastanum L..*	(A) Increased bleeding [Hu et al. 2005]	–	–
*Carthamus tinctorius L.*		(B)Potentiates its activity Lim et al. [99]	(B) Potentiate prolongation of bleeding time and prothrombin time Xiao et al. [103]; (B) potentiates its activity Lim et al. [99]
*Curcuma longa L.*	(B) PAF inhibitor Leite et al. [92]	(A) COX-inhibitor Lim et al. [99]	–
*Ginkgo biloba L.*	(A) Inhibiting CYP2C9/C19, CYP3A4, CYP1A2 Costache et al. [109] (B) Additive effect [Hu et al. 2005]; (B) PAF receptor antagonist Leite et al. [92]		
*Panax ginseng Meyer*	(B) Additive effect [Hu et al. 2005]	(B) Inhibited platelet aggregation Lim et al. [99]	
*Salvia miltiorrhiza*	(A) Increased bleeding; (B) additive effect [Hu et al. 2005]	(B) Additive or synergistic effect Lim et al. [99]	
*Zingiber officinale Roscoe*	(B) PAF inhibitor Leite et al. [92]		

(A) pharmacokinetic interaction, (B) pharmacodynamic interaction

### Safety profile

Salvia miltiorrhiza, Angelica sinensis (Oliv.) Diels and Zingiber officinale Roscoe were identified to cause major interactions with anticoagulant or antiplatelet drugs may lead to life-threatening complications or serious adverse events (Tsai et al. [110]).

## Conclusions

In this review, extensive search has been done on herbal sources investigated for anti-thrombotic activity recently were highlighted. Adverse haemorrhagic complications due to current conventional medicines, patient safety, huge economic burden on healthcare, cognisance of herbal drug interaction, and complications due to recently emerged pandemic due to SARS Co-V2 virus, etc. all pose a need to search for newer pharmacological moieties for drug discovery.

## Data Availability

Data sharing not applicable to this article as no data sets were generated or analyzed during the current study.

## Abbreviations

CVD: Cardiovascular disease
CAD: Coronary artery disease
PAD: Peripheral arterial disease
VTE: Venous-thrombo-embolic disease
NHANES: The National Health and Nutrition Examination Survey
CHD: Coronary heart disease
AHA: American Heart Association
COVID-19: Coronavirus disease 2019
SARS Co-V-2: Severe acute respiratory syndrome coronavirus 2
CAC: Covid-19-associated coagulopathy
ICU: Intensive care unit
ARDS: Acute respiratory distress syndrome
ADR: Adverse drug reaction
NSAID: Non-steroidal anti-inflammatory drug
TT: Triple therapy
DAPT: Dual antiplatelet therapy
DT: Dual therapy
PCI: Percutaneous coronary intervention
IHD: Ischaemic heart disease
AF: Atrial fibrillation
WHO: World Health Organization
HDI: Herb-drug interaction
UDP: Uridine di-phosphate
CYP: Cytochrome
